# Diurnal variation in Uchikomi fitness test performance: Influence of warm-up protocols

**DOI:** 10.3389/fpsyg.2022.1059727

**Published:** 2022-11-17

**Authors:** Özgür Eken, Fatma Hilal Yagin, Ismihan Eken, Tomasz Gabrys, Vera Knappova, Serdar Bayrakdaroglu, Zeki Akyildiz, Hadi Nobari

**Affiliations:** ^1^Department of Physical Education and Sport Teaching, Inonu University, Malatya, Turkey; ^2^Department of Biostatistics and Medical Informatics, Faculty of Medicine, Inonu University, Malatya, Turkey; ^3^Department of Physical Education and Sport, Faculty of Education, University of West Bohemia, Pilsen, Czechia; ^4^Movement and Training Science, Gumushane University, Gumushane, Turkey; ^5^Sports Science Department, Gazi University, Ankara, Turkey; ^6^Faculty of Sport Sciences, University of Extremadura, Cáceres, Spain; ^7^Department of Motric Performance, Transilvania University of Brasov, Brasov, Romania

**Keywords:** diurnal variation, combat sports, specific testing, judo, martial arts

## Abstract

Performance is judged using a variety of methods to ensure uniformity between competitions. Uchikomi Fitness Test (UFT) could accomplished between morning qualifying and evening finals. The purpose of this study is to investigate the impact of different warm-up protocols on UFT at different times of the day in female judokas. Ten volunteer women who had been practising judo on a regular basis for more than 5 years and actively competed in international tournaments took part in this study. Judokas completed UFT, either after no-warm-up (NWU), specific warm-up (SWU), and linear+lateral warm-up (FWU) protocols for two times a day in the morning: 09:00–11:00 and in the evening: 16:00–18:00, on non-consecutive days. In conclusion, there was a significant increase in UFT scores (*F* = 9.89; *p* = 0.002), a + b (*F* = 4.42; *p* = 0.04) and heart rate (*F* = 28.99; *p* < 0.001) in the early evening compared to the morning. Increases in UFT performance were observed in the SWU protocol compared to the NWU and FWU protocols (*p* < 0.05). However, the interaction between time of day and warm protocol was not significant (*p* > 0.05). The UFT performance revealed diurnal variation, and the judokas’ performances may be favourably affected more in the late hours, particularly following SWU procedures.

## Introduction

Judo is high-intensity intermittent grappling Olympic combat sport that requires high physical conditioning, and technical-tactical excellence for success in competition (Franchini et al., 2008, 2013; Hamdi et al., 2013). The duration of a judo match is 4 min excluding extra-time (i.e., when the match is tied during the regular time it continues up to the golden score or up to one of the athletes is disqualified). During a judo match energy is supplied mainly by the oxidative system, but the scoring actions are supported by the anaerobic pathways (Julio et al., 2017). Judokas require a great deal of aerobic and neuromuscular strength to complete the wide variety of motions and movements with sufficient intensity to defeat their opponent (Detanico et al., 2012; Tack, 2013).

Judokas must be in peak physical and mental condition at all times because of the rapid nature of the fights. One of the methods to be applied to begin the competition at the greatest level and important for the preparation for the judo match in the competition is the ideal warm-up plan, which judokas need neuromuscular preparation for the competition (Miarka et al., 2011). It’s possible to take a variety of approaches in the lead-up to a judo bout. One promising strategy involves using post-activation potentiation to prime the neural readiness for subsequent powerful actions (Lum, 2019). Another option is to use a high-load inspiratory muscle warm-up, which has not been shown to be useful in simulated judo tests (Merola et al., 2019). Alternately, leg strength may be affected by dynamic stretching that increases mobility (Eken et al., 2020). Other hypotheses include normal jogging or running, as well as combat-based activities (Lum, 2019).

In order to determine the effects of different warm-up protocols on official competitions, researchers can use a number of specific judo tests that may provide some reference as an alternative method (Lum, 2019; Merola et al., 2019). One of the well-accepted tests to simulate judo demands is the Uchikomi Fitness Test (UFT; Almansba et al., 2007, 2012; Kons et al., 2021). UFT is a valid and reliable test to predict the performance of judokas in different training periods, as it was associated with both neuromuscular effort and cardiorespiratory adaptations and meeting the muscle strength and cardiovascular adaptations specific to judo (Almansba et al., 2011, 2012). Also, Almansba et al. reported that UFT was physiologically similar to the energy expended during judo competition (Almansba et al., 2012). In the literature, there are various research focusing at UFT performance (Almansba et al., 2007). Between elite and non-elite athletes, Almansba et al. (2007) found no difference in the total number of attempts or the total number of attempts in the best two phases (Almansba et al., 2007). Additionally, Ceylan and Balcı (2018) observed no similarity between the physiological reactions (lactate accumulation and heart rate) indicated after structured judo matches and UFT in their study (Ceylan and Balcı, 2018). According to Kons et al. (2018), anaerobic power and lower extremity strength performance are connected to UFT performance (Kons et al., 2018).

There is no research in the existing body of literature that suggests that the performance of the UFT is affected by the various warm-up methods. There are a few studies that can be found in the research literature that investigate how different warm-up routines affect performance on the specific judo fitness test (SJFT), which is one of the various judo performance tests (Lum, 2019; Eken et al., 2022). Using SJFT as a reference test, a study compared different warm-up protocols (based on post activation potentiation vs. conventional) revealing the beneficial effects of post activation potentiation on performance and peak power (Lum, 2019). One study that used another SJFT as a reference test compared the effect of different warm-up protocols on diurnal variation (specific warm-up vs. dynamic warm-up), and the results showed that time of day and warm-up protocols have significant effects on SJFT performances (except for post-warm HR protocols and index, which do not have significant effects; Eken et al., 2022). On the other hand, there are still a great deal of studies that need to be carried out in order to examine the various effects that several warm-up regimens can have on performance during SJFT and UFT. Additionally, the effects of warm up can be limited by the time of day in which they are performed. The time of day is also a significant factor because of its connection to the diurnal variation, as well as the biochemical and hormonal responses to this pattern. As an example, judoka’s heart rate, blood lactate, body temperature and rate of perceived exertion seems to be significantly higher in the evening than in the morning (Öztürk et al., 2022). In the other study, judoka’s muscle power and strength seems to be significantly higher in the afternoon than in the morning (Chtourou et al., 2013). Naturally, the quantity and quality of sleep as well as athletic performance may operate as mediators or moderators for such changes; yet, it appears that the time of day is crucial for ultimate physical and physiological performance in judo as well as in other sports (Thun et al., 2015).

While additional research on the impact of various warm-up protocols on UFT performance in judokas is required due to the dearth of relevant literature, it is possible that time of day should be regarded a significant factor in determining whether or not such an effect exists. In addition, it is extremely important for judokas to combine the most appropriate time of day with the most appropriate warm-up protocol in terms of reaching maximum performance both before training and competitions. Therefore, this study has a twofold purpose. One of the purposes is to analyze the performance of different warm-up protocols on UFT. Another aim is to explore possible interactions of different warm up protocols with time of day. It is hypothesized that the addition of Specific warm up (SWU) will result in increased UFT results as compared to FWU and NWU. It is also hypothesized that evening results will be better as compared morning results.

## Materials and methods

### Participants

Ten women volunteers (mean age: 18.5 ± 1.0 years-old, mean height: 161.6 ± 4.5 cm, mean body mass: 62.2 ± 9.1 kg, HR_rest_: 60.80 ± 6.47 bpm), who regularly participated in judo training for more than 5 years. Besides volunteers were black belts (belt level of the judokas were 1st DAN) and actively competed in international competitions, participated in this study. The UFT groups were categorized in such a way that their weights were close to each other [mean body mass: 50.00 ± 2.0 kg (52 kg-48 kg-50 kg together) and 67.43 ± 3.9 kg (65 kg-63 kg-65 kg-67 kg-70 kg-75 kg-67 kg together)]. Because three judo athletes, two partners to be thrown (uke) and one executant (tori) of the same weight category, were required to participate in this test. These judokas were not in the competitive phase or rapid weight loss process. Judokas reported having no problems with insomnia or anxiety.

The power analysis program G*Power (version 3.1.9.3, Germany) was used to obtain the research group. Theoretical power analysis was performed using the “ANOVA: Repeated measures within-between interaction” test (alpha value = 0.05 and test power (1-beta value) = 0.80, partial eta squared ($\eta_{p}^{2}$) = 0.30 and repeated correlation between measurements = 0.70). As a result, it was reported that at least 9 female judoka should be included in the study (Faul et al., 2007). Before starting the study, judokas were informed in detail about the content, purpose and methodological model of the research. The judokas completed an Informed Consent Form, stating that they volunteered to take part in the training, and it was submitted to the appropriate authorities. Inonu University Clinical Research Ethics Committee gave its approval to each and every test and measurement that was carried out as part of this investigation (Approval Number: 2021/2172). While ten of the judokas competed actively in international competitions. All athletes were proficient in the ippon-seoi-nage and sode-tsuri-komi-goshi technique and had been engaging in resistance training twice a week for at least a year. Before the study, judokas were asked to night sleep at least 8 h before each test session, and to come on a full stomach, provided that they had food at least 2 h before the morning and early evening session. The study was conducted 4 weeks after the national championships (December-2021). The participants were given necessary information about maintaining their usual judo training, not doing high-intensity exercise, and not using substances such as alcohol and caffeine (Reilly et al., 2007). The inclusion criteria were as follows: (1) active and regular participation in all phases of the study, (2) not suffer any disease or injury that could affect the results, (3) prior to the tests, participants were not allowed to do additional exercises, such as high-intensity exercise and high-intensity resistance training, other than their own judo training, so as not to affect the test results.

### Study design

**Figure 1:**
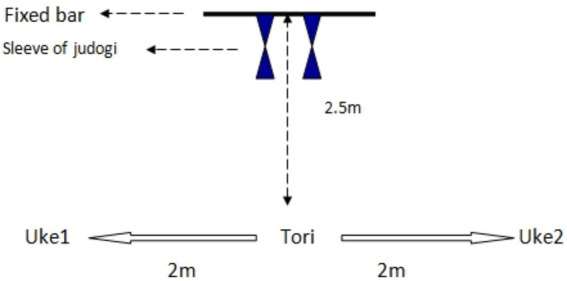
Uchi-komi Fitness Test schematic representation (Almansba et al., 2007).

The resting heart rate of the judokas were measured by themselves using the Polar RS400 as soon as they got out of bed in the morning, without speaking or moving. The Uchikomi Fitness Test (UFT) performance of the participants was measured after different warm-up protocols [No-warm-up (NWU), Specific warm up (SWU), Linear and Lateral warm up (FWU)] in two different time periods of the day (between 9:00 am and 11:00 a.m. and 4:00 pm and 6:00 p.m) with at least 2 days between each other (Souissi et al., 2013). The judokas used to train regularly regardless of the time they participated in the study between 09:00–11:00 a.m. and 04:00–06:00 p.m. They were training 2 days apart in the week. The times of day used in the study design were the same. Thus, it was hoped to ascertain the extent to which the standardized design, which was created prior to the study, had an effect on the competition. No extra training program except research protocol was applied to the judokas during the familiarization phase and the continuation of the experimental design. The Karvonen formula was used to calculate the heart rate reserve (HRR) in order to determine the running intensity of the judokas individually in the general warm-up phase before each test session. The warm up intensity was defined by the target heart rate using the formula by Karvonen as follows: target heart rate = exercise intensity × (maximum heart rate − resting heart rate) + resting heart rate (Karvonen et al., 1957; Nes et al., 2013). Polar RS400 brand watch was used to monitor heart rate during 5 min of jogging, and after UFT performance. The study consisted of 3 sessions, subjects were randomly assigned in a counterbalanced manner to either perform the NWU (only 50% of HRR, 15 min. jogging), SWU (50% of HRR, 5 min jogging +10 min. Judo-specific warm-up exercise), and FWU (50% HRR, 5 min jogging +10 min. Linear and lateral warm-up exercise) routine before performing the UFT performance. UFT schematic representation is shown in Figure 1.

#### Warm-up protocols

##### No warm up

The rate of the warm up was determined by calculating each judoka’s 50% HRR (Karvonen et al., 1957). The judokas did only 15 min of light jogging under the control of experts. Thus, both warm-up intensity and warm-up differences between the judokas in the training were eliminated. After 15 min’ light jogging, judokas’ UFT were performed.

##### Specific warm up

According to the 50% HRR result of each judokas, the judokas did only 5 min of light jogging under the control of experts (Karvonen et al., 1957). Judokas performed SWU exercise after light jogging. This warm up consisted of 11 SWU (foot sweeps, finger wrist and ankle rotations, trunk side stretch, trunk rotator stretch, hip circles, knee bends, cartwheels both sides, forwards rolls, backwards rolls, forward rolls with legs spread and backwards roll with legs spread) exercises (Table 1). They performed all SWU exercise totally 10 min.

**Table 1:** SWU protocol.

SWU	Description
Foot sweeps (1 m work – 1 m rest)	Moving side to side, sweep foot along floor.
Finger, wrist, and ankle rotations (30 s work – 30 s rest)	Rotate ankles and wrists to stretch flexors and extensors.
Trunk side stretch (30 s work – 30 s rest)	Standing, lean to one side then the other with arms overhead.
Trunk rotator stretch (30 s work – 30 s rest)	Standing, rotate body from side to side.
Hip circles (30 s work – 30 s rest)	On all fours, circle hip inside body and away from body, switch.
Knee bends (30 s work – 30 s rest)	Bouncing from kneeling position to standing position, stretch hamstrings.
Cartwheels both sides (30 s work – 30 s rest)	Standing facing the side, cartwheel.
Forwards rolls (15 s work – 15 s rest)	Standing, perform a forward roll into side body landing.
Backwards rolls (15 s work – 15 s rest)	Standing, perform a backward roll.
Forward rolls with legs spread (15 s work – 15 s rest)	Same forward roll with spread legs.
Backwards roll with legs spread (15 s work – 15 s rest)	Same backwards roll with spread legs.

##### Linear + lateral warm up (FWU)

The rate of the warm up was determined by calculating each judoka’s 50% HRR (Karvonen et al., 1957). After 5 min jogging, judokas performed FWU exercise. FWU exercise consisted of stationary spider-man (30 s work – 30 s rest), inchworm (30 s work – 30 s rest), backward and forward lunge walks [2× (30 s work – 30 s rest)], backpedal (30 s work – 30 s rest), straight-leg skip (30 s work – 30 s rest), heel-ups [2× (30 s work – 30 s rest)] and high knee run [2× (30 s work – 30 s rest)] (Boyle, 2004). They performed all FWU exercise totally 10 min.

### Methodology

Body weights were determined using an electronic scale (Tanita SC-330S, Tanita, Netherlands) that had an accuracy of 0.1 kilos when measuring judokas (kg). A precision stadiometer with a reading resolution of 0.01 meters was used to measure the height of the judokas who volunteered (SECA-Mod.220, Seca GmbH & Co. KG., Germany; American College of Sports Medicine, 2018).

**Figure 2:**
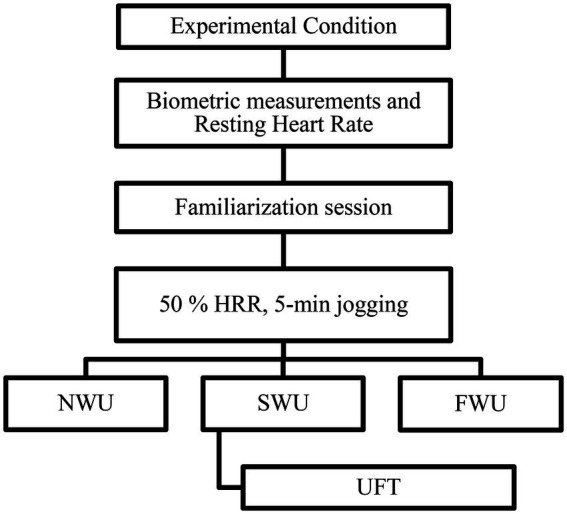
Experimental design.

UFT is an intermittent test specific to judo, and last 243 s in total duration. This test requires three judo athletes in the same weight category, two athletes to be thrown (uke), and one to execute the throws (tori). Almansba et al., developed the UFT to assess the efforts of the athletes during the judo matches in terms of both qualitative (compliance with various stages observed in the matches), and quantitative (effort-pause relationship). In both early evening and morning hours, the judokas did the uchi-komi exercise consisting of 6 levels during the test, and executed the isometric chin-up in the judogi mounted on a fixed place within the specified time. At each stage, the duration of the uchi-komi was 20 s. The isometric chin-ups in gripping the judogi were increased by 3 s after rests at each level and lasted from 6 s to 18 s. Rests lasted from 4 s to 12 s, increasing by 2 s at each level. The judokas performed the techniques with maximum intensity and accuracy. The sequence of work was as follows: isometric posture of the upper extremity (holding tightly, grasping): the athlete (tori) fixed her hands, and hung on the judogi on the bar; b) dynamic and explosive phase: the athlete got out of the the horizontal bar where she was hung, and began to execute the uchi-komi in each of the two athletes (uke) positioned 4 m apart from each other, using two techniques: ippon-seoi-nage (arm technique) and sode-tsuri-komi-goshi (hip technique). The intensity was controlled by the signal. The total number of uchi-komi performed, the number in the best two stages (a + b) was counted, and the athlete’s HR were recorded by Polar RS400 watch throughout the test (Almansba et al., 2007). Experimental design is shown in Figure 2.

### Statistical analysis

The conformity of quantitative data to normal distribution was evaluated with the Shapiro–Wilk test for univariate analyzes and the Henze Zirkler test for multivariate analyses. While demographic data were summarized with mean and standard deviation because they showed normal distribution, UFT tests and heart rate data were summarized with median and interquartile range (IQR) because they did not show normal distribution. In the UFT, both the total number of uchikomi performed correctly in all six sets and the sum of the scores corresponding to the two best-performing sets (a + b) were recorded. During UFT, a Polar RS400 is used to capture the user’s HRmax and HRmean (min - 1 pulse count) readings. The HRmean / HRmax ratio was indexed as %HRmax. Two-Way Permutation Analysis of Variance (PERMANOVA) test was used to compare two differently measured UFT results (a + b, total scores, and heart rate responses) with the first factor being the three warm-up protocols (NWU, SWU, and FWU) and the second-factor being time of day (morning and early evening). In the PERMANOVA analysis based on Euclidean similarity matrices, the residuals were performed with a reduced model with 9,999 permutations. The significance level was interpreted according to *p* < 0.05. Data analyzes were performed using Python 3.9 and IBM SPSS Statistics 28.0 for Windows (New York; USA).

## Results

**Figure 3:**
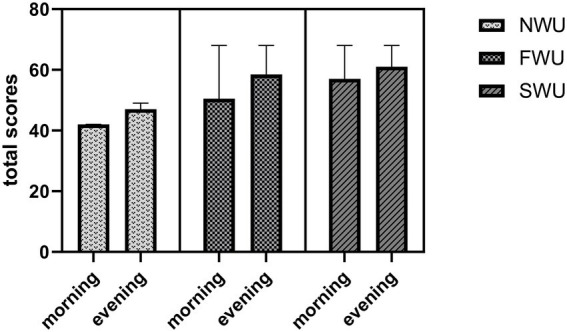
Uchi-komi Fitness Test (total scores) in the morning and evening hours for the three warm-up protocols. NWU, No-warm-up; SWU, Specific warm up; FWU, Linear and Lateral warm up.

Figure 3 shows the changes in the UFT (total scores) values of the participants. According to the results of the study, there was a statistically significant difference between the measurements in terms of UFT (total scores; *F* = 9.89; *p* = 0.002). In addition, a statistically significant difference was found between the warm-up protocols in terms of UFT (total scores; *F* = 31.65; *p* < 0.001). *Post hoc* analyzes showed that there was a statistically significant difference in UFT (total scores) between NWU and SWU (*p* < 0.001), NWU and FWU (*p* < 0.001) and SWU and FWU (*p* = 0.006) groups. According to the data obtained in the study, the interaction effect was not found statistically significant (*F* = 0.87; *p* = 0.42).

**Figure 4:**
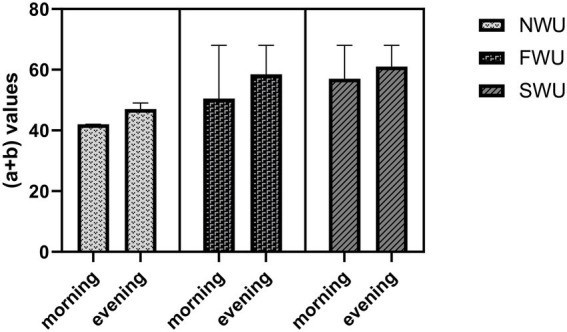
Uchi-komi Fitness Test (a + b) in the morning and evening hours for the three warm-up protocols. NWU, No-warm-up; SWU, Specific warm up; FWU, Linear and Lateral warm up.

Time of day had the main effect on UFT a + b repetitions and these values were higher in the early evening than in the morning (*F* = 4.421; *p* = 0.04). In addition, according to the findings of the study, warm-up protocols for UFT a + b repetitions had a main effect (*F* = 22.40; *p* < 0.001). Post-hoc analyzes showed that the UFT a + b repeats values were significantly higher in the SWU group compared to the NWU and FWU groups (*p* < 0.001). However, there was no interaction between warm-up protocols and time of day for UFT a + b repetitions (*F* = 0.561; *p* = 0.563; Figure 4).

**Figure 5:**
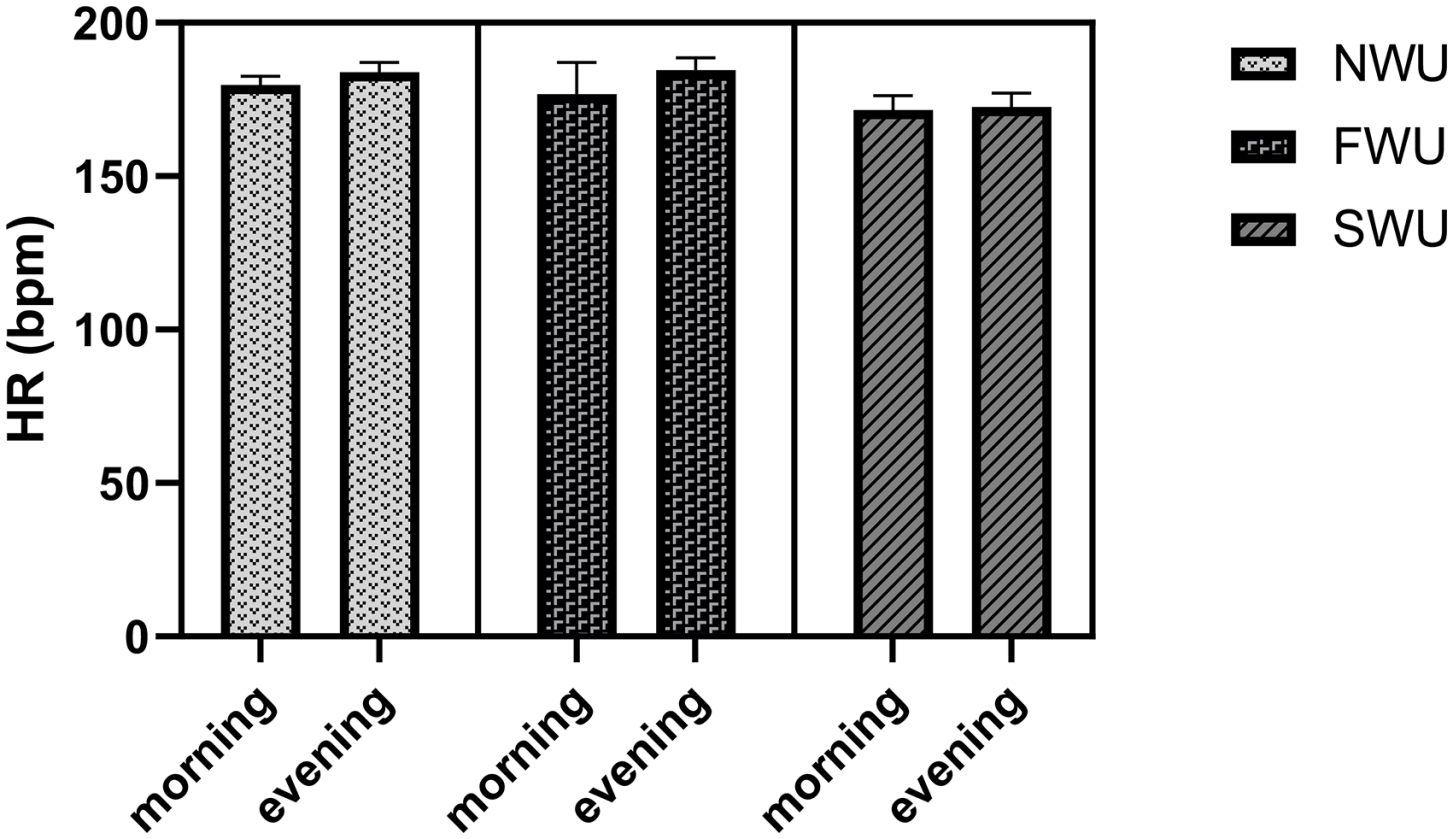
The heart rates of participants in the morning and evening hours for the three warm-up protocols. HR, Heart rate; NWU, No-warm-up; SWU, Specific warm up; FWU, Linear and Lateral warm up.

Figure 5 shows the changes in participants’ average HR values during the UFT. According to the findings of the study, there was a statistically significant difference between the measurements in terms of average HR (*F* = 28.99; *p* < 0.001). In addition, a statistically significant difference was found between the warm-up protocols in terms of average HR (*F* = 21.15; *p* < 0.001). *Post hoc* analyzes showed that there was a statistically significant difference in average HR between the NWU and FWU (*p* = 0.001), NWU and SWU (*p* = 0.09), and SWU and FWU (*p* = 0.002) groups. The interaction effect of warm-up protocols and time of day for average HR was statistically significant (*F* = 33.77; *p* < 0.001).

## Discussion

This study aims to determine the impact of different warm-up protocols (NWU, SWU, and FWU) performed at different times of the day (morning and early evening) on UFT performance. According to the results of the study, the participants’ Uchi-komi Fitness Test (total scores) values measured in the morning were significantly lower than in the evening. In other words, it was observed that the exercise performed in the evening increased the Uchi-komi Fitness Test (total scores). In addition, the Uchi-komi Fitness Test (total scores) value was significantly higher in the SWU protocols compared to the NWU and FWU protocols. Based on this, it can be said that the SWU protocols positively affects the Uchi-komi Fitness Test (total scores). However, time * warm-up protocol interaction was not statistically significant for Uchi-komi Fitness Test (total scores). Participants’ Uchi-komi Fitness Test (a + b) values were significantly higher in the evening than in the morning. In addition, it was observed that the SWU protocols increased the Uchi-komi Fitness Test (a + b) value. The time * warm-up protocols interaction was not significant for the Uchi-komi Fitness Test (a + b). The participants’ average HR in the evening hours was significantly lower compared to the morning hours. In addition, the average HR value was lower in the SWU protocols compared to the FWU and NWU protocols. The time * warm-up protocol interaction was significant for average HR. These findings were found to be consistent regardless of the time of day. When the findings of the study were analyzed, it was revealed that the research’s hypotheses had been proven correct. When it comes to planning the training for a sport, it is essential to have a thorough knowledge of the optimal working time for both the body and the mind. This is because performance time is an essential component of every activity (Atkinson and Reilly, 1996).

No matter whether of the three warm-up protocols was used, the results of this study revealed that there was a significant increase in UFT in the a + b and total scores in the early evening compared to the morning. Our findings take on an added level of significance given that there has not been a previous study that measured the effect that the time of day had on the UFT performance of judo athletes. On the other hand, there are studies that determine the effect that different times of the day have on the various performance parameters of judo athletes. According to the findings of Chtourou et al. (2018), the repeated sprint running performance and mood of the elite athletes who were examined did not show a significant influence on the time of day the test was performed. According to what he reported, this finding may be attributable to the practice of working out first thing in the morning (Chtourou et al., 2018). Chtourou et al. (2013) investigated the effect of time of day on short-term maximum performances before and after a judo match in young judo athletes. According to the findings of the research, judo athletes had much more muscle strength and power in the afternoon than they had in the morning. This difference was statistically significant. After judo competitions, researchers discovered that these daily variations were reversed and associated with greater levels of weariness in the afternoon than in the morning (Chtourou et al., 2013). An increase in body temperature as a result of diurnal variation may be a reflection of passive muscle warming, and it may also cause an increase in the rate of metabolic reaction, an increase in the extensibility of connective tissue, a decrease in the viscosity of muscle, and an increase in the rate at which action potentials are conducted (Racinais et al., 2017). Additionally, the variation that occurs in one’s body temperature during the day might lead to improved motor coordination, which in turn can lead to increased performance in the afternoon as opposed to the morning (Lericollais et al., 2009). These diurnal improvements in muscle performance have been reported to be due to enhanced muscle contraction properties rather than a change in neural drive modification in the evening. In order to understand why there is a diurnal variation in performance, it is necessary to first determine the cause of this variation (Racinais et al., 2005). In additional research involving the circadian rhythm, long-term and short-term performance of exercise was examined (Chtourou et al., 2013). According to these investigations, lactic acid levels, heart rate, and anaerobic power were higher in the afternoon and evening than in the morning. (Ünver and Atan, 2021) In this study, the fact that the UFT was higher in the early evening hours than in the morning hours may be associated with a higher increase in body temperature due to warm-up protocol.

Special warm-ups should be discipline specific. These exercises can affect the activation of performance-limiting muscles that are directly related to sports. In this way, nerve processes are stimulated, muscles are toned and there is an increase in tension. Increased muscle activation can reduce elastic and viscous resistances in the muscle through warm up (Bishop, 2003). However, although not significant, greater increases in morning to early evening in UFT performance (total scores) were observed in the SWU group. When heart rate was used as a criterion for recovery, significant declines were found in the judo athletes’ heart rates following both the morning and evening UFT tests. This demonstrates that they are recovering well. No studies on the influence of warm-up methods on the UFT test in Judo have been undertaken. In addition, the literature regarding functional warm-up and judo-specific warm-up procedures for sports performance is scarce (Boyle, 2004; Hammerel, 2012). When the effect of the warm-up and stretching procedures on the time of day was investigated, it was determined that early morning performances of performance agility, 505 changes of direction (CoD), 10 meter sprint, and early evening performances were better than the change of direction open test (CoDD; Ben Maaouia et al., 2020; Kerdaoui et al., 2021). Previous studies reported that the upper and lower body (ULB) warm up protocols before Judo Specific Fitness Test increased performance (Lum, 2019), and also static stretching improved the flexibility, and static stretching after dynamic warm up increased the leg force (Eken et al., 2020). Hammerel (2012) reported that static stretching significantly decreased Judo Specific Fitness Test index, and did not affect heart rate, and throw with technique performance (Hammerel, 2012). It’s possible that reflex inhibition is the cause of the increase in flexibility that comes from static stretching. A prolonged increase in elastic range of motion (ROM) can be the consequence of several factors, including an enhanced strain tolerance, a decrease in viscoelasticity, and, to some extent, a reduction in muscle-external stiffness (Kay et al., 2015). The fact that mutual inhibition occurs for a considerable amount of time after stretching and contributes to viscoelastic and morphological changes is the mechanism that produces an increase in leg strength after doing warm-up protocols that include static stretching and dynamic stretching (Behm, 2018).

The findings of this study are subject to certain restrictions. The performance of the UFT was assessed twice throughout the day at different time intervals. In this particular study, the afternoon hours were not analyzed, nor were the menstrual cycle phases of the female athletes who participated taken into consideration. In this study, the effect of different hours and heating status was investigated. In future research, more comprehensive protocol designs can be made by minimizing the limitations. In addition, the researchers in this study did not take the time to record the individuals’ core temperatures. In addition, the fact that the participants were not judokas competing in a single weight category is another limitation of the study. One of the other limitations of the study is the number of judo athletes. In future research, the study can be revised to include more judo athletes. In addition to these, the subject group in the study consisted of only female judokas. Repeating the study with a larger number of male and female judo players who are considered to be of elite and top-elite status over a range of ages is possible. Increasing the number of studies examining the effects of different warm-up protocols and time of day effect on different performance parameters in Judo (Uchikomi Fitness Test, Judo Specific Fitness Test, Santos Test etc.) may give more some specific recommendations about the planning of judo-specific warm-up applications before training programs. This can contribute to judo athletes getting maximum efficiency from their judo performance both before training and competitions and minimizing the risk of injury.

## Conclusion

The present study revealed that there was time of day effect on UFT performance. According to that, UFT performance (total scores) increased in the early evening hours compared to the morning hours. In addition, the Uchi-komi Fitness Test (total scores) value was significantly higher in the SWU protocols compared to the NWU and FWU protocols. In addition, the average heart rate value was lower in the SWU protocols compared to the FWU and NWU protocols. SWU protocol is a warm-up that imitates judo techniques and is associated with the characteristic structure of judo. Also, increased performance with SWU in the early evening may have triggered a further increase in UFT performance. The high UFT performance after SWU protocol, although not significant, may be related to this explanation. In sum, the results of the present study suggest that SWU protocol is sufficient to alter UFT performance in positive way in the early evening hours compared to morning hours. This can be taken into account when planning training programs.

## Data availability statement

Publicly available datasets were analyzed in this study. This data can be found at: https://zenodo.org/record/7117724#.Yzgu9C3OnBI.

## Ethics statement

The studies involving human participants were reviewed and approved by Inonu University Clinical Research Ethics Committee (Approval Number: 2021/2172). The patients/participants provided their written informed consent to participate in this study.

## Author contributions

ÖE, FHY, IE, TG, VK, SB, ZA, and HN: conceptualization, methodology, and writing—review and editing. ÖE, FHY, IE, SB, and HN: data curation. ÖE, FHY, TG, VK, SB, and ZA: formal analysis. TG and VK: funding acquisition. ÖE, TG, ZA, and HN: supervision. ÖE, FHY, IE, TG, SB, ZA, and HN: writing—original draft. All authors contributed to the article and approved the submitted version.

## Funding

Published with the financial support of the European Union, as part of the project entitled development of capacities and environment for boosting the international, intersectoral, and interdisciplinary cooperation at UWB, project reg. no. CZ.02.2.69/0.0/0.0/18_054/0014627.

## Conflict of interest

The authors declare that the research was conducted in the absence of any commercial or financial relationships that could be construed as a potential conflict of interest.

## Publisher’s note

All claims expressed in this article are solely those of the authors and do not necessarily represent those of their affiliated organizations, or those of the publisher, the editors and the reviewers. Any product that may be evaluated in this article, or claim that may be made by its manufacturer, is not guaranteed or endorsed by the publisher.
